# Non-canonical BAD activity regulates breast cancer cell and tumor growth via 14-3-3 binding and mitochondrial metabolism

**DOI:** 10.1038/s41388-018-0673-6

**Published:** 2019-01-11

**Authors:** Jasdeep Mann, John Maringa Githaka, Timothy W. Buckland, Ning Yang, Rachel Montpetit, Namrata Patel, Lei Li, Shairaz Baksh, Roseline Godbout, Hélène Lemieux, Ing Swie Goping

**Affiliations:** 1 Department of Biochemistry, University of Alberta, Edmonton, AB T6G 2H7 Canada; 2 Department of Oncology, University of Alberta, Edmonton, AB T6G 2H7 Canada; 3 Department of Pediatrics, University of Alberta, Edmonton, AB T6G 2H7 Canada; 4 Department of Medicine, University of Alberta, Edmonton, AB T6G 2H7 Canada; 5 Faculty Saint-Jean, University of Alberta, Edmonton, AB T6G 2H7 Canada

**Keywords:** Breast cancer, Cancer metabolism, Tumour biomarkers, Mechanisms of disease, Cell signalling

## Abstract

The Bcl-2-associated death promoter BAD is a prognostic indicator for good clinical outcome of breast cancer patients; however, whether BAD affects breast cancer biology is unknown. Here we showed that BAD increased cell growth in breast cancer cells through two distinct mechanisms. Phosphorylation of BAD at S118 increased S99 phosphorylation, 14-3-3 binding and AKT activation to promote growth and survival. Through a second, more prominent pathway, BAD stimulated mitochondrial oxygen consumption in a novel manner that was downstream of substrate entry into the mitochondria. BAD stimulated complex I activity that facilitated enhanced cell growth and sensitized cells to apoptosis in response to complex I blockade. We propose that this dependence on oxidative metabolism generated large but nonaggressive cancers. This model identifies a non-canonical role for BAD and reconciles BAD-mediated tumor growth with favorable outcomes in BAD-high breast cancer patients.

## Introduction

The BH3-only protein BAD has multiple functions that are determined by cellular context. Apoptotic roles in lymphocyte development and metabolic roles in pancreatic glucose sensing have been clearly defined through physiological studies of genetic engineered mice [1–4]. *Bad* null animals had diminished apoptotic signalling and developed late-onset lymphomas [1, 5], as well as diminished glycolysis with altered glucose homeostasis [2]. Phosphorylation of BAD was critical to both of these phenotypes through cell-specific signaling. In developing B and T cells, phosphorylation of S155 (homologous to S118 in humans) within the BH3 domain inhibited apoptosis by preventing BAD binding to anti-apoptotic Bcl-2, and protection from mitochondrial outer membrane permeabilization [1]. In pancreatic cells, phosphorylated BAD was bound to the regulatory glycolytic enzyme glucokinase and stimulated catalytic activity necessary for insulin release and maintenance of circulating glucose levels [3, 6]. Other tissues affected by in vivo genetic manipulation of *Bad* were neurons and isolated mammary gland epithelial cells that showed alterations in both metabolism and apoptosis [4, 5].

Given that altered apoptosis is a hallmark of malignancy and cancer progression [7], multiple studies have identified associations between apoptotic regulators and clinical disease. In line with this, BAD is differentially expressed in human cancers of the ovary [8], lung [9], colon [10] and breast [11–13]. We showed that in primary breast cancer, elevated BAD levels correlated with a 3.7-fold increased likelihood of patient survival and was a better prognostic indicator than tumor grade, HER2 or estrogen receptor suggesting a causal contribution to tumor suppression [13]. Surprisingly, BAD did not sensitize breast cancer cells to apoptosis but instead stimulated progression through the cell cycle. Thus, the role of BAD in breast cancer and how this relates to clinical outcome is unclear. In order to explore this, we examined the effect of BAD on breast cancer cells and identified unexpected mechanisms regulating cell growth. We found that BAD regulated breast cancer cell growth by concurrent phosphorylation dependent and independent pathways. BAD phosphorylation drove cellular growth and tumor aggressiveness. BAD also regulated mitochondrial oxidative metabolism, independent of phosphorylation status. These studies identify novel BAD signaling pathways in breast cancer that may give insight to clinical outcomes.

## Results

### BAD regulates cell growth

**Fig. 1:**
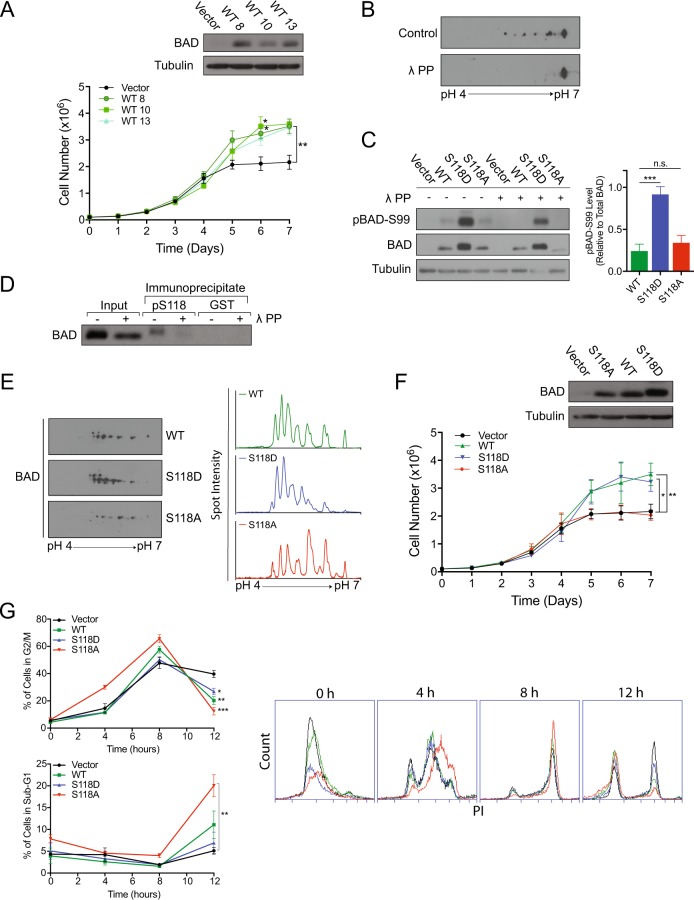
BAD expression increases cell number. **a** Top: Western blot analysis of MDA-MB-231 cells expressing pcDNA3.2-V5-DEST vector control or multiple clones of WT-BAD. Below: Cell count assay over 7 days (error bars ± SEM of 3 independent experiments). **b** 2D immunoblot of BAD expressing cells treated with λ protein phosphatase or phosphatase inhibitor (control) and probed with BAD antibody. **c** Left: Immunoblots of indicated cell lines treated with phosphatase inhibitor (-) or λ protein phosphatase (+) probed with antibodies against BAD, pBAD-Ser99 and tubulin. Right: Graphs of mean protein quantitation (error bars ± SEM of 5 independent experiments). **d** Phosphorylated BAD at S118 was immunoprecipitated from MDA-MB-231 BAD-expressing lysates, treated with λ protein phosphatase ( + ), or phosphatase inhibitor (-) and immunblotted against total BAD. GST antibody was used as a negative control. **e** Left: 2D immunoblot of WT-, S118D- and S118A-expressing cell lines probed with total BAD antibody. Right: Histograms depicting spot intensity of 2D immunoblot normalized to background levels. **f** Top: Immunoblot of indicated cell lines probed for BAD. Bottom: Cell count assay over 7 days (error bars ± SEM of 3 independent experiments). **g** Left: Quantification of G2/M population (top) and sub-G1 population (below) at indicated time points collected via flow cytometry at FL-2 channel (error bars ± SEM of 3 independent experiments). Right: Histograms of DNA content (FL-2) at indicated time points

To investigate the effect of BAD on breast cancer growth, we generated cell lines expressing BAD to characterize growth effects and gain mechanistic insight. MDA-MB-231 cells that have low endogenous BAD expression were stably transfected to express ectopic BAD [13]. Cells were grown in culture for 7 days without media change to mimic tumor-like conditions and cell counts were recorded (Fig. 1a). Vector control cells showed the expected cellular accumulation and reached a plateau by day 5. BAD-expressing cells, on the other hand, showed extended and increased cellular accumulation dependent on ectopic BAD expression (Supplementary Fig. 1A). To validate this result with loss-of-function studies, BAD expression was knocked out in mammary epithelial MCF10A cells, which express higher levels of endogenous BAD (Supplementary Fig. 1B). Loss of BAD inhibited cell accumulation demonstrating that this effect was not cell-line restricted (Supplementary Fig. 1C). Together, these results demonstrated that BAD expression supported cell growth.

### BAD phosphorylation regulates cell and tumor growth

To investigate mechanism, we queried whether phosphorylation of BAD regulated this growth effect. In particular, BAD apoptotic activity is attenuated by phosphorylation of three key serine residues (S75, S99 and S118) in response to survival signaling [14–16]. We first assessed BAD phosphorylation in these cells. In 2D-IEF/SDS western blotting, BAD migrated as multiple isoforms that collapsed to a single spot in response to phosphatase treatment (Fig. 1b), indicating the presence of multiple phosphorylated forms of BAD. Phospho-specific antibodies confirmed that a proportion of these isoforms were phosphorylated at S99 and S118 (Fig. 1c, d). Since phosphorylation of S118 regulates binding to Bcl-XL and apoptotic and metabolic activities of BAD [2, 16–18], we directly tested the contribution of S118 phosphorylation on cell and tumor growth. We generated cell lines stably expressing phosphomimetic (S118D) or non-phosphorylatable (S118A) mutants of BAD. Relative to BAD-expressing cells, BAD-S118A-expressing cells showed a lower proportion of spots in the acidic range, whereas BAD-S118D-expressing cells showed increased proportion of acidic spots (Fig. 1e). BAD-S118A-expressing cells showed decreased growth properties to BAD-S118D-expressing cells, indicating that phosphorylation enhanced cell growth. (Fig. 1f). Cell synchronization revealed that ectopic BAD expression, irrespective of phospho-status, stimulated earlier exit out of G2/M compared to vector cells (Fig. 1g). Notably, S118A-expressing cells had significantly increased sub-G1 population compared to vector, which is consistent with the lower accumulation of S118A-expressing cells in the cell count assay. These results confirmed that the BAD growth effect was due to increased cell cycle dynamics and differential apoptosis.

**Fig. 2:**
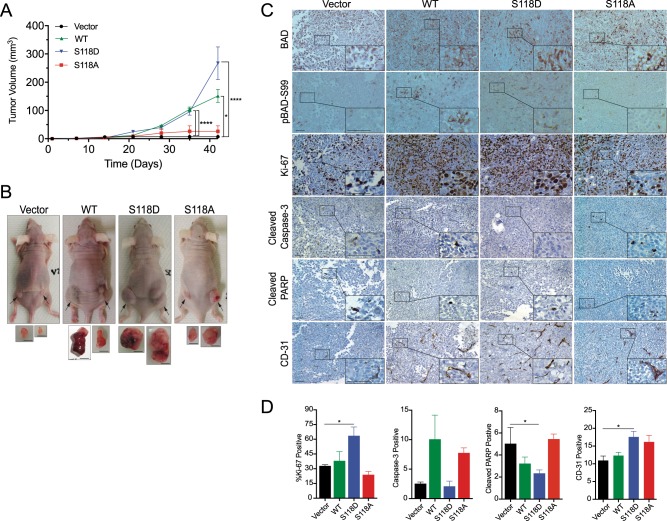
BAD expression increases tumor growth. **a** Mean tumor volume of indicated MDA-MB-231 xenograft tumor ± SEM (vector *n* = 4, WT-BAD *n* = 4, BAD-S118D *n* = 6, and BAD-S118 *n* = 4). **b** Representative images of endpoint animals and excised tumors. Black arrows indicate tumor location. Scale bar = 0.5 cm. **c** Representative immunohistochemistry of subcutaneous tumors stained with indicated antibodies. Scale bar = 50 μm. **d** Quantification of mean immunohistochemical staining from 10 random fields of view per individual tumor. Ki-67-positive cells were quantified as a percentage of total cells per field of view. Cleaved caspase-3, cleaved PARP, and CD-31-positive cells were quantified as the absolute number of positive cells per field of view (error bars ± SEM)

We next tested whether BAD also regulated growth in a tumor-bearing model. We injected wild-type and mutant BAD-expressing cells into the subcutaneous flanks of mice and measured tumor volume over time. BAD-S118D-expressing cells formed the largest tumors and wild-type BAD-expressing cells formed intermediate sized tumors, which were both significantly larger than tumors from BAD-S118A- and vector control cells (Fig. 2a, b). S118D-derived tumors were generally solid whereas wild-type BAD-derived tumors had larger necrotic centers, suggesting that tumor growth in these two genotypes occurred via different molecular pathways (Supplementary Figure 2). To query pathways that contributed to differential tumor size, we examined proliferation and apoptosis markers in the xenograft tumor tissue (Fig. 2c, d). BAD-S118D-expressing tumors had significantly increased Ki67-positive and CD-31 positive cells, supporting that increased proliferation and angiogenesis contributed to tumor growth. Additionally, these tumors had decreased cleaved PARP levels, suggesting that decreased apoptosis contributed to larger tumor volume. On the other hand, tumors that expressed wild-type BAD shared characteristics between BAD-S118A or S118D tumors. Overall, enforced phosphorylation mimic of BAD at S118 drove tumor growth associated with increased proliferation, angiogenesis and decreased apoptosis.

### BAD phosphorylation-mediated growth requires 14-3-3 binding

**Fig. 3:**
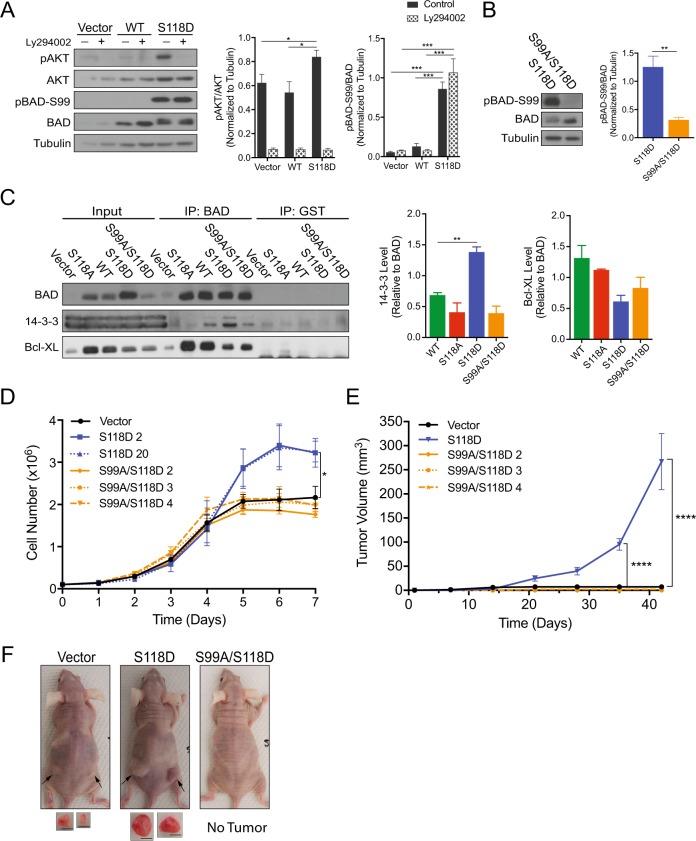
BAD S99 phosphorylation is required for S118D-mediated cell and tumor growth. **a** Left: MDA-MB-231 cells expressing pcDNA3.2-V5-DEST vector control, WT-BAD, or BAD-S118D were grown normally for 3 days prior to 50 μM Ly294002 addition for 1 h prior to cellular lysis. Right: Quantification of protein band density (error bars ± SEM of 3 independent experiments). **b** Left: Immunoblot depicting BAD protein levels in indicated cell lines. Right: Quantification of protein band density (error bars ± SEM of 3 independent experiments). **c**–**f** MDA-MB-231 cells expressing pcDNA3.2-V5-DEST vector control, BAD-S118D, BAD-S118A, and BAD S99A/S118D. **c** MDA-MB-231 cells expressing the indicated mutations were subjected to immunoprecipitation by BAD or GST (control) antibodies and immunoblotted against BAD, 14-3-3, and Bcl-XL. Levels of 14-3-3 and Bcl-XL binding were compared to BAD IP levels (error bars ± SEM of 3 independent experiments). **d** Cell count assay over 7 days (error bars ± SEM of 3 independent exkperiments). **e** A total of 3 × 10^6^ cells of vector, BAD-S118D, or BAD-S99A/S118D, were injected into the subcutaneous flanks of nude mice. Tumor volume was measured weekly for 7 weeks (vector *n* = 4, BAD-S118D *n* = 6, BAD-S99A/S118D clone 2 *n* = 6, clone 3 *n* = 4, clone 4 *n* = 5; error bars ± SEM). **f** Representative images of subcutaneous tumor growth of nude mice taken at day 42. Black arrows indicate tumor location. Images of tumors are depicted below. Scale bar = 0.5 cm

To decipher the BAD phosphorylation-dependent pathway, we further examined the effect of BAD-S118D on cell growth. We noted that S118D-expressing cells had a higher proportion of phosphorylated isoforms of BAD (Fig. 1e), including phosphorylation at S99 (Fig. 1c). Phosphorylation of S99 can be mediated by AKT [15] and is critical for 14-3-3 binding and attenuation of BAD apoptotic activity [15, 19–21]. BAD-S118D-expressing cells showed increased AKT activity compared to vector and WT cells, suggesting that BAD-S118D-expression enhanced survival signaling (Fig. 3a). AKT inhibition by the PI3K inhibitor Ly294002 did not attenuate pS99-BAD levels, indicating AKT does not phosphorylate BAD at S99 in our model. Phosphorylated S75 and S99 are bound by 14-3-3 [17, 22–24] and we reasoned that BAD-S118D mediated cell growth and survival through binding to 14-3-3. We mutated S99 to alanine within the S118D background (BAD-S99A/S118D) and examined 14-3-3-interaction (Fig. 3b). As expected, 14-3-3 bound to BAD-S118D and this binding was abrogated in the double mutant BAD-S99A/S118D (Fig. 3c). Importantly, the double mutation of S99A/S118D reduced the cellular growth rate to background vector levels, indicating that S118D-mediated growth was dependent on downstream phosphorylation of S99 (Fig. 3d). To test whether phosphorylation of S99 alone was sufficient to drive increased cell proliferation, we individually expressed single mutants BAD-S99D and BAD-S99A (Supplementary Fig. 3A). Neither of these mutants supported elevated cell proliferation likely due to their inability to bind 14-3-3 (Supplementary Fig. 3B), again suggesting that 14-3-3 binding to BAD enhanced cell growth. When injected into recipient mice, S99A/S118D-derived tumors showed no growth advantage and were indistinguishable from vector control cells (Fig. 3e, f). Therefore, the enhanced cellular and tumor growth imparted by BAD-S118D was dependent on S99 phosphorylation and associated with 14-3-3 binding and resistance to apoptosis. These results indicated that phosphorylation of BAD at S118 stimulated survival pathways that in turn phosphorylated BAD at S99 resulting in binding to 14-3-3 proteins.

### Wild-type BAD mediates growth through a phosphorylation-independent mechanism

**Fig. 4:**
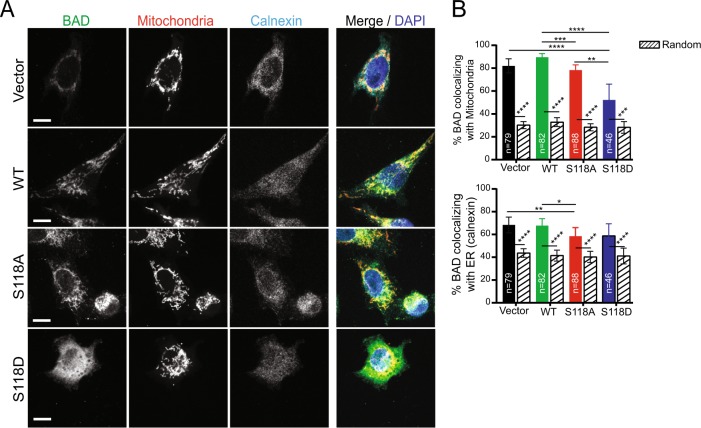
Wild-type BAD co-localizes strongly with mitochondria. **a** Z slice of confocal imaging of MDA-MB-231 cells expressing pcDNA3.2-V5-DEST vector control, WT-BAD, BAD-S118D or BAD-S118A immunostained with antibodies against BAD (green) and calnexin (endoplasmic reticulum (ER) marker) (cyan). MitoTracker™ Red and DAPI were used as mitochondria (red) and nuclei (blue) markers, respectively. Scale bar = 10 μm. **b** Percentage of BAD protein (BAD channel MCC fraction converted to percentage) co-localizing with mitochondria (top) and ER (bottom) (colored bars) compared to randomized control (patterned bars) (*n* = number of cells; Kruskal-Wallis test)

**Fig. 5:**
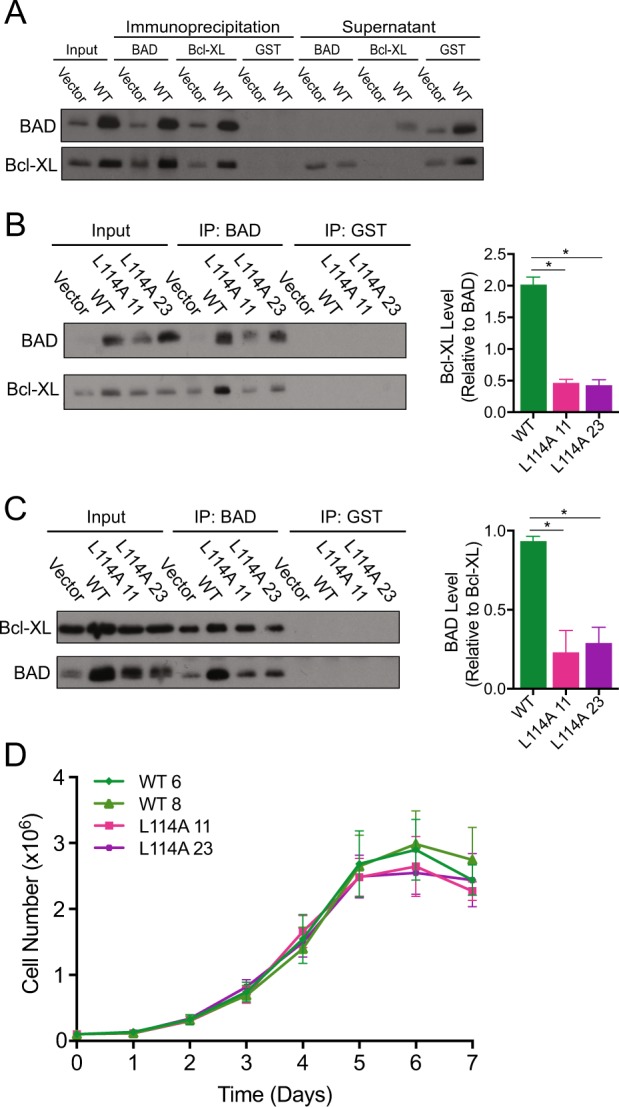
The BAD:Bcl-XL interaction is not required for BAD-mediated growth. **a** MDA-MB-231 cells expressing pcDNA3.2-V5-DEST vector control or WT-BAD were subjected to immunoprecipitation (IP) by BAD, Bcl-XL, and GST (control) antibodies. Supernatant (SN) of IPs were retained. IP and SN were subjected to protein analysis via western blot and immunoblotted for BAD and Bcl-XL. **b**–**d** MDA-MB-231 cells expressing pcDNA3.2-V5-DEST vector control, WT-BAD, and BAD-L114A (2 clones). **b**, **c** Cells were subjected to immunoprecipitation by either BAD, Bcl-XL, or GST (control) antibody and probed for BAD and Bcl-XL antibody on western blot. Relative binding levels were quantified (error bars ± SEM of 3 independent experiments). **d** Cell count assay over 7 days (no significance; error bars ± SEM of 3 independent experiments)

Wild-type BAD stimulated cell and tumor growth that was driven by different pathways than S118D. Unlike BAD-S118D, wild-type-BAD was minimally phosphorylated at S99 (Fig. 1c), did not increase AKT activity (Fig. 3a), and bound poorly to 14-3-3 (Fig. 3c). Additionally, wild-type BAD was predominanqtly located at the mitochondria, while BAD-S118D cells showed a different staining pattern with significantly less mitochondrial localization, again supporting different mechanisms for cell growth stimulation (Fig. 4a, b). Given that wild-type BAD bound strongly to Bcl-XL (Fig. 3c), we asked whether increased growth was mediated through a Bcl-XL-dependent pathway. We first assessed the proportion of wild-type BAD that was bound to Bcl-XL. We performed co-immunoprecipitation assays and compared the levels of BAD that were bound or not bound to Bcl-XL (Fig. 5a). Anti-Bcl-XL antibodies immunoprecipitated the majority of BAD from cell lysates, indicating that most of BAD was bound to Bcl-XL. While BAD:Bcl-XL binding liberates pro-apoptotic Bax/Bak to trigger cell death [25, 26], this was inconsistent with our observed increased cell/tumor volume and lack of apoptotic markers. Instead, in other contexts, BAD:Bcl-XL complexes that release BAX can indirectly trigger cell cycle entry into G1 [27]. Therefore, we hypothesized that expression of ectopic wild-type BAD sequestered excess Bcl-XL to stimulate cell proliferation.

To interrogate this, we generated a BAD-L114A mutant that disrupts Bcl-XL binding [28]. Co-immunoprecipitation experiments confirmed that the L114A mutants had diminished binding to Bcl-XL (Fig. 5b, c). If the BAD:Bcl-XL interaction was critical for the growth phenotype, BAD-L114A mutants would show decreased growth. However, the growth profile of these mutants was similar to wild-type BAD (Fig. 5d), indicating that the BAD:Bcl-XL interaction was dispensable for growth. Thus, wild-type BAD stimulated cell and tumor growth through an alternative pathway that did not require S118 phosphorylation, 14-3-3 binding or Bcl-XL interactions.

### BAD increases mitochondrial metabolism

**Fig. 6:**
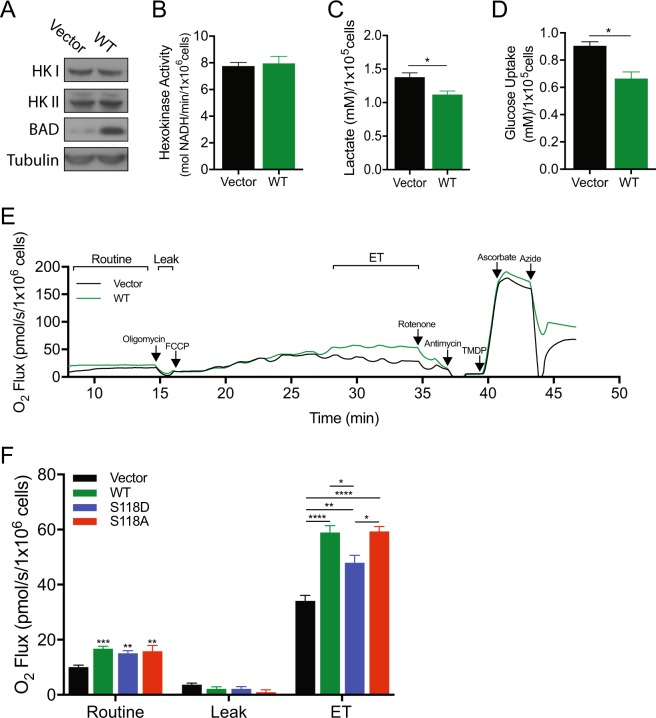
BAD promotes mitochondrial metabolism. **a**–**d** MDA-MB-231 cells expressing pcDNA3.2-V5-DEST vector control or WT-BAD. **a** Immunoblot measuring Hexokinase I and II levels. **b** Hexokinase activity (absorbance) of cells was measured at 490 nM and normalized to cell number (no significance; error bars ± SEM of 3 independent experiments). **c**, **d** Cells were grown for 96 h (**c**) or 48 h (**d**) prior to medium collection and addition of lactate oxidase (**c**) or glucose oxidase (**d**). **d** Fluorescence was measured at 590 nM and normalized to cell number (error bars ± SEM of 3 independent experiments). **e**, **f** MDA-MB-231 cell lines expressing pcDNA3.2-V5-DEST vector control, WT-BAD, BAD-S118D or BAD-S118A were grown in normal growth conditions for 3 days prior to high-resolution respirometry analysis. **e** Representative trace of high-resolution respirometry in intact cells. Oxygen consumption is plotted as a function of time. Arrows indicate times of titrations. **f** Bar graph representing the results of the various states measured with high-resolution respirometry (error bars ± SEM of 6 independent experiments)

In addition to regulating apoptosis, BAD also modulates other cellular processes. In liver and pancreatic cells, BAD binds to glucokinase (hexokinase IV) near the active site to stimulate enzymatic activity and indirectly increase mitochondrial metabolism [2, 6]. It is unknown if this activity is relevant in our studies since breast cancer cells do not express the tissue-restricted glucokinase [29]. Nevertheless, these cells do express the related hexokinases I and II [30, 31]. Therefore, we examined whether BAD modulated cellular metabolism, and consequently, survival and proliferation of breast cancer cells. To test the role of wild-type BAD in metabolism, we measured hexokinase activity, lactate production and glucose uptake as markers of glycolysis. Elevated BAD levels did not alter hexokinase enzyme levels or activity (Fig. 6a, b), indicating that unlike liver and pancreatic cells, BAD did not appear to stimulate the first regulatory step of glycolysis. BAD-expressing cells however, did show altered glycolytic flux and produced significantly less lactate and took up less glucose compared to vector control cells (Fig. 6c, d), suggesting that BAD increased mitochondrial oxidative metabolism. We directly assessed oxidative metabolism with high-resolution respirometry and observed that BAD expression significantly increased cellular oxygen consumption (Fig. 6e, f). Inhibiting mitochondrial electron flow with the ATP synthase inhibitor oligomycin blocked oxygen uptake, confirming that oxygen consumption was via mitochondrial complex IV (Fig. 6f, Leak). When we depolarized the mitochondria with the uncoupler FCCP to measure maximal electron transport (ET) activity, BAD-expressing cells recovered increased oxygen consumption indicating that BAD stimulated electron flow through the ET (Fig. 6f, ET). BAD-induced stimulation of mitochondrial respiration did not require phosphorylation of S118 as both the WT-BAD and S118A mutants showed elevated oxygen consumption relative to vector control (Fig. 6f). However, the S118D mutant showed significantly less oxygen consumption relative to wild-type BAD expressing cells, but was still significantly higher than vector cells. We postulate that BAD-mediated stimulation of mitochondrial metabolism contributes, in part, to cell proliferation. Interestingly, while S118A increased ET and early G2/M exit out of the cell cycle, S118A also stimulated apoptosis (Fig. 1g) possibly accounting for its decreased overall cell number in the cell count assay.

To further query mechanisms of this oxidative effect and since BAD:Bcl-XL interactions are known to stimulate mitochondrial ATP production [32], we measured oxygen uptake in the presence of the BH3-mimetic ABT-737. ABT-737 decreased BAD:Bcl-XL interactions as expected, but did not diminish oxygen consumption (Supplementary Fig. 4). Therefore, BAD increased oxygen consumption through a novel mechanism that was downstream of glycolysis and was not dependent on S118 phosphorylation or Bcl-XL binding.

### BAD increases complex I activity

**Fig. 7:**
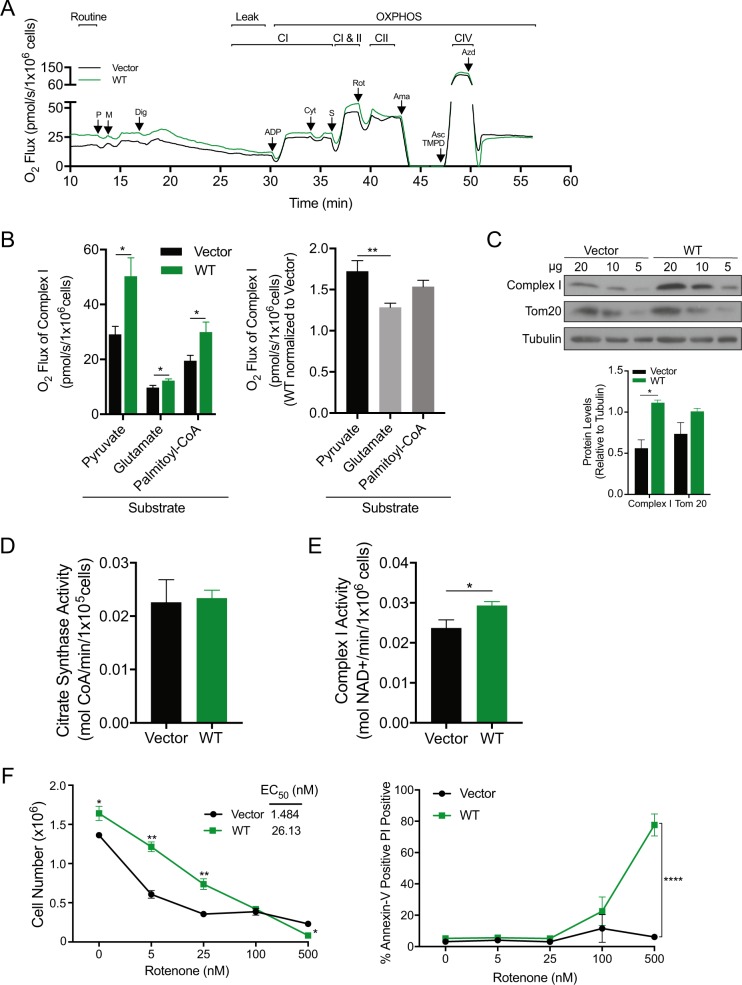
BAD increases complex I activity of the ETC. **a**, **b** MDA-MB-231 cell lines expressing pcDNA3.2-V5-DEST vector control and WT-BAD were grown normally in complete growth conditions for 72 h prior to respirometry analysis. **a** Representative experiment showing oxygen flow as a function of time. Respiration is expressed in pmol oxygen per second per million cells. Arrows indicate the times of titrations. Abbreviations used: pyruvate (P), malate (M), digitonin (Dig), adenosine diphosphate (ADP), cytochrome *c* (Cyt), succinate (S), rotenone (Rot), antimycin A (Ama), tetramethyl-phenylenediamine (TMPD), ascorbate (Asc), and azide (Azd). **b** Graphical representation of oxygen flux of complex I activity with the indicated substrates (error bars ± SEM of minimum 5 independent experiments). **c** MDA-MB-231 cell lines expressing pcDNA3.2-V5-DEST vector control and WT-BAD were probed for complex I and Tom20 levels. Decreasing concentrations of protein were analyzed (paired student’s *t*-test at 20 μg concentration; error bars ± SEM of 3 independent experiments). **d**-**e** MDA-MB-231 cell lines expressing pcDNA3.2-V5-DEST vector control and WT-BAD. **d** Citrate synthase activity was measured at 412 nM and normalized to cell number (error bars ± SEM of 3 independent experiments). **e** Complex I activity was measured at 540 nM and normalized to cell number (error bars ± SEM of 4 independent experiments). **f** Cells were delivered the indicated concentrations of rotenone for 5 days prior to flow cytometric analysis with Annexin V-647/PI staining. Left: Living cell number was determined by gating the healthy cell population using BD Accuri C6 software. EC_50_ was determined using GraphPad Prism software. Right: The Annexin V-647 positive/PI positive population is graphed (error bars ± SEM of 3 independent experiments)

Electrons enter the ET from either complex I (CI) or complex II (CII), converge at complex III (CIII) and are delivered to the site of oxygen reduction in complex IV (CIV). To dissect how BAD increased electron flow, we examined the dependence on specific ET complexes. We permeabilized the plasma membrane with digitonin in the absence of exogenous substrates to obtain baseline oxygen consumption rates (Fig. 7a, Leak). Vector and BAD-expressing cells had similar leak respiration rates indicating that BAD did not increase electron flow by increasing permeability of the inner membrane (Fig. 7a, Leak). Upon addition of substrates pyruvate/malate and ADP, BAD-expressing cells showed elevated oxygen consumption relative to control cells (Fig. 7a, CI). CII activity was measured by the subsequent addition of the CII substrate succinate with concomitant inhibition of CI with rotenone. This was necessary to isolate CII from CI activity, as both deliver electrons to CIII and CIV. BAD did not increase CII activity (Fig. 7a, CI vs. CII). Thus, BAD increased mitochondrial oxidative metabolism by stimulating electron flow through CI. Notably, pyruvate is one of the last metabolites produced in glycolysis meaning that BAD functioned downstream of glycolysis.

To examine whether BAD had a direct effect on mitochondria, we queried whether BAD-stimulated oxygen consumption was substrate-dependent. Carbons derived from different nutrient sources enter the mitochondria through different carriers and are converted to metabolites that converge at the TCA cycle to generate NADH and FADH_2_ that then donate electrons to the ET. Carbohydrates, amino acids and fatty acids deliver carbon to the mitochondria in the form of pyruvate, glutamate and palmitoyl-CoA, respectively. We had already determined that BAD increased oxidative metabolism of pyruvate, so we next measured oxygen consumption derived from glutamate or palmitoyl-CoA (Fig. 7b). BAD-expression increased CI activity regardless of carbon source suggesting increased activity at or downstream of the TCA (Fig. 7b). However, when comparing oxygen consumption between fuel sources, BAD-expressing cells showed the greatest oxygen consumption with pyruvate versus either palmitoyl-CoA or glutamate suggesting a fuel use preference. To assess whether BAD increased mitochondrial metabolism or increased mitochondrial biomass, we measured levels of the mitochondrial marker Tom20 [33] and citrate synthase activity. We found no difference between vector and BAD expressing cells, indicating that increased oxygen consumption was likely not related to increased mitochondrial biomass (Fig. 7c, d). Interestingly, BAD-expressing cells had increased levels of the 20 kDa subunit (iron-sulfur protein 7) of CI (Fig. 7c). We therefore directly measured CI enzyme activity in vitro (Fig. 7e). Wild-type BAD cells increased CI activity compared to vector, thus suggesting a mechanism by which BAD promoted mitochondrial mechanism. In conclusion, BAD stimulated ET activity and oxygen consumption through a novel mechanism.

To test the effects of increased mitochondrial metabolism, we inhibited CI activity and assessed cell growth. CI contributes to cellular proliferation [34] and rotenone-treated vector cells showed a dose-dependent decrease in cellular accumulation as expected (Fig. 7f left). BAD-expressing cells were more tolerant of rotenone effects with an EC_50_ that was approximately 15 times higher than that of vector control cells. Further, BAD-expressing cells were dependent on CI activity for survival, as these cells underwent apoptosis at high concentrations of rotenone (500 nM), whereas control cells were cytostatic, but did not die (Fig. 7f right). Possibly, this dependence on oxidative metabolism contributed to the necrotic centers we observed in BAD-expressing tumors (Supplementary Fig. 2). These results suggested that although BAD-expressing tumors were significantly larger than vector-derived tumors, they may not necessarily predict aggressive disease. Since tumors with high proportion of cancer stem cells are associated with aggressive disease [35], we assessed cancer stem-like properties through mammosphere assays. BAD expression did not affect the mammosphere-efficiency but decreased mammosphere area (Supplementary Fig. 5), suggesting that increased tumor volume was not driven by elevated cancer stem cells. Thus, BAD-expressing cells induced elevated oxygen consumption that facilitated cell growth and also sensitized to cell death in response to CI-blockade.

**Fig. 8:**
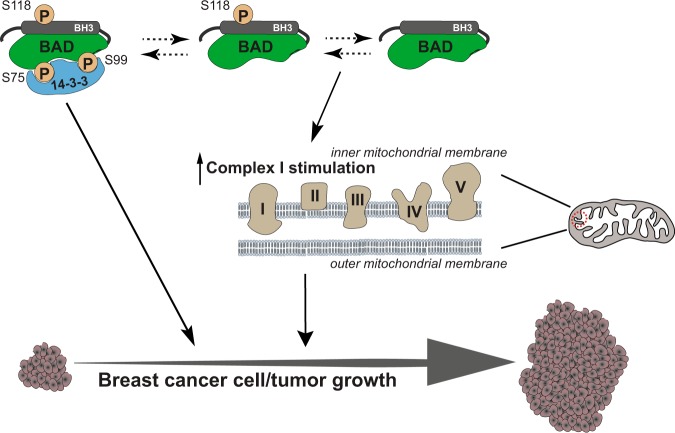
Schematic representation of BAD function in breast cancer growth and survival. BAD phosphorylation at S118 increases binding to 14-3-3 proteins which enhances breast cancer cell and tumor growth. Additionally, BAD stimulates complex I activity of the electron transport chain in the mitochondria to regulate cell survival

In summary, our data demonstrated that BAD increased cellular accumulation resulting in increased tumor volume (Fig. 8). Phosphorylation of BAD at S118 contributed to this phenotype by stimulating proliferative and survival pathways. Concurrently, BAD stimulated oxidative catabolism of carbohydrate, fatty acid and amino acid nutrient sources through a mechanism involving CI activation and oxygen consumption. Inhibition of BAD-mediated CI stimulation demonstrated a causal link between mitochondrial metabolism and BAD-regulated cell growth and survival.

## Discussion

We show here that BAD increased the growth of breast cancer cells in culture and in a tumor-bearing model. Specifically, BAD increased cell cycle dynamics in association with mitochondrial localization and increased mitochondrial complex I oxidative metabolism. Enhanced mitochondrial metabolism did not require phosphorylation of regulatory S118, and was supported not only by glucose, but also by fatty acid and amino acid carbon sources, altogether pointing to a novel molecular mechanism. Phosphomimic S118D mutants additionally contributed to cell growth effects by stimulating survival and decreasing apoptotic pathways dependent on 14-3-3 binding, respectively. Thus, BAD increases breast tumor growth through two distinct pathways that can be differentiated by dependence on S118 phosphorylation.

Similarly, Smith et al. found an increase in prostate cancer cell number and tumor growth as a result of ectopic BAD expression [36]. BAD has been previously shown to increase cell cycle progression in fibroblasts through bypassing G0/G1 growth arrest via binding to Bcl-XL and Bcl-2 [27, 37]. Our studies, however, indicated that BAD:Bcl-XL interactions were dispensable as expression of mutant BAD-L114A cells maintained enhanced growth. Instead, enforced phosphorylation of BAD at S118 drove an aggressive growth phenotype that was dependent on S99 phosphorylation and binding to 14-3-3. This growth mechanism was similar to other studies where BAD phosphorylation at S118 enhanced cellular survival by inhibiting BAD:Bcl-XL interaction [16], while phosphorylation at S99 promoted BAD binding to 14-3-3 proteins and attenuated apoptosis [17, 24]. In line with this, BAD phosphorylation promoted gastric cancer cell survival in response to growth factors [38, 39]. Thus, forced expression of a BAD mutant with a phosphomimic at S118 can drive breast cancer cell and tumor growth. The physiological contexts where this is occurring however, is unclear as the majority of BAD was dephosphorylated in this model system.

Notably, we identified a novel growth-stimulating pathway that was dependent on BAD expression but not reliant on phosphorylation. BAD-expressing cells enhanced carbon catabolism preferentially from carbohydrates, but also from fatty acid and amino acid sources to elevate mitochondrial oxidative metabolism, which contributed to cell growth. Studies in mouse hepatocytes and pancreatic β-cells identified that BAD phosphorylation at S155 (homologous to S118 in human) specifically affected glucose metabolism by interacting with and stimulating glucokinase [2, 6, 40]. Ablation of BAD phosphorylation at S155 in primary cultures of cortical neurons and astrocytes significantly blunted their oxygen consumption rate [4]. In breast cancer cells of this study, however, BAD-mediated changes in glucose metabolism were downstream of glycolytic hexokinase and independent of phosphorylation status. BAD did not increase lactate production or glucose uptake; markers of increased glycolysis [41] and instead, increased mitochondrial oxygen consumption. Further, this effect was also induced by amino acid and fatty acid catabolism. Thus, this novel mechanism whereby BAD alters cellular carbon flux may be through direct mitochondrial effects.

How BAD-enhanced oxidative metabolism contributes to cell growth is likely complex. Oxidative metabolism opposes the Warburg effect whereby rapidly proliferating cells, including cancer cells, take up glucose and secrete lactate in the presence of oxygen, as a means to produce precursors for macromolecular synthesis [42]. However, cancer cells do maintain oxidative metabolism to some level, even in the face of increased glycolysis [34] as mitochondrial metabolism has also been shown to facilitate cell and tumor growth. For example, defects in mitochondrial respiration in renal tumors prevented progression as a result of p53 and AMPK activation [43]. Additionally, mitochondrial reactive oxygen species (ROS) production was required for Kras-mediated regulation of cell proliferation and tumorigenesis [44] and loss of mtDNA with subsequent loss of mitochondrial respiration in HEK293 cells significantly impaired cellular proliferation [45]. In line with this, we found BAD-mediated CI activation facilitated cellular proliferation since cells were resistant to growth inhibition by low doses of rotenone.

Although the wild-type BAD expressing cells grew more robustly in culture and formed large tumors compared to vector control, they were not more aggressive. BAD-expressing tumors did not show increased vascularization, which is a marker of metastasis. Their necrotic centers likely reflected increased sensitivity to stress conditions inherent in the tumor environment. Further, BAD decreased glycolysis, whereby aerobic glycolysis has been shown to drive metastasis in part through upregulation of HIF and SNAIL [46]. By instead promoting oxidative metabolism, BAD may skew tumor characteristics toward less aggressive cancers, as we identified BAD expression reduced the size of mammospheres, a marker for the stem cell population. BAD stimulated CI-mediated mitochondrial metabolism and inhibition of CI induced apoptosis specifically in BAD-expressing cells, suggesting an addiction to CI activity and possible increased sensitivity to stressors such as ROS. This understanding of BAD-mediated metabolic effects may yield new insights into cancer therapeutics. For example, the CI inhibitor and diabetic drug metformin was associated with decreased cancer risk [47] indicating potential application for BAD-high breast cancers.

In summary, we have identified a novel mechanism of BAD-mediated growth in breast cancer. BAD, located at the mitochondria, can stimulate cell cycle progression through increased oxidative phosphorylation and CI activity. Additionally, this metabolic effect generates non-resilient tumors and suggests a mechanism whereby BAD promotes cell growth while predicting favorable outcomes in clinical breast cancer.

## Materials and methods

### Cell culture and reagents

MDA-MB-231 and MCF10A cell lines were from ATCC (Manassas, VA, USA). Cells were cultured as described previously [13]. Cell lines were routinely tested negative for mycoplasma contamination using the MycoSensor Series PCR Assay Kit (Agilent Technologies, Santa Clara, CA, USA). Cells were passaged maximum 25 times after resuscitation. λ protein phosphatase was from New England Biolabs (Ipswich, Massachusetts, USA), Ly294002 was from Cell Signaling Technologies (Danvers, Massachusetts, USA), and ABT-737 was from Santa Cruz Biotechnology (Santa Cruz, California, USA).

### Stable cell line creation

Human BAD cDNA was generated using Superscript III Reverse Transcriptase Polymerase Chain Reaction (Invitrogen, Carlsbad, CA, USA). Primers (IDT, Coralville, IA, USA) used are listed in Supplementary Table S1. BAD cDNA was cloned into the pcDNA3.2/V5-DEST vector (Invitrogen). BAD mutations were created using QuikChange Lightning Site-Directed Mutagenesis Kit (Stratagene, San Diego, CA, USA).

### Cell count assay

A total of 1 × 10^5^ cells were plated, in duplicate, in 60 mm tissue culture dishes and cells were counted in duplicate as for the time period indicated.

### Protein analysis

A total of 2D gel electrophoresis was carried as previously described [48]. pH 4–7 gel strip and 12% SDS-PAGE were used for first and second dimensional electrophoresis, respectively. Further experimental details are described in Supplemental Materials and Methods.

Methodology for immunoblotting, co-immunoprecipitation and flow cytometry were as done previously [13].

### Cell synchronization to G1/S boundary and DNA content analysis

Cell synchronization at G1/S by double thymidine block was similar to [49]. Briefly, cells were plated in 12-well plates at 25% confluency for 24 h prior to the initial addition of thymidine (2 mM) for 16 h. Cells were then released for 8 h prior to the second thymidine block (2 mM) for 16 h. Cells were then released and allowed to cycle through the cell cycle and harvested and fixed at the indicated time points prior to propidium iodide (Invitrogen) staining (0.1% Triton X-100, 2 mg/mL RNase, 20 μg/mL propidium iodide). Flow cytometry was performed using the BD Accuri^TM^ C6 to analyze DNA content in the FL-2 channel.

### Mouse studies

Animal procedures were performed in compliance with the Canadian Council on Animal Care and approved by the Institutional Animal Care and Use Committee (AUP00000386). Cells were injected into subcutaneous flanks of nude mice at a concentration of 3 × 10^6^ cells in 100 μL matrigel/media mix (1:3 ratio). RPMI 1640 medium (Life Technologies, Carlsbad, CA, USA) with no supplemental serum or antibiotic and BD Matrigel Matrix was used (BD 354234, Mississauga, ON, CAN). Tumor volume was measured weekly for 7 weeks. Tumor volume (mm^3^) was calculated as follows: (length × width × height)/2.

### Immunohistochemistry

Immunohistochemistry was similar to [50]. Antigen retrieval was performed in 10 mM sodium citrate, 0.05% Tween 20, pH 6.0 for antibodies to pBAD-S99, BAD, Ki-67, cleaved PARP, and CD-31 or 10 mM Tris base, 1 mM EDTA, 0.05% Tween 20, pH 9.0 for cleaved caspase-3. Images were taken with a Zeiss AxioObserver Z1 Microscope at ×20 with 1.6X Optovar with ZEISS ZEN imaging software. Ki-67 quantification was performed using ImmunoRatio software [51] that uses a color deconvolution algorithm to calculate percentage of positively stained nuclei. Cleaved caspase-3, CD-31, and cleaved PARP were quantified manually per field of view. Antibody information can be found in Supplemental Materials and Methods.

### Immunofluorescence and confocal imaging

Cells were grown on coverslips in 24 well plates. Cells were pre-incubated with 200 nM MitoTracker™ Red (Invitrogen) for 15 min, followed by 10 min fixation with 4% PFA (ThermoFisher Scientific). For intracellular staining, cells were permeabilized with 0.1% Triton X-100 in PBS for 10 min and blocked with 0.3% BSA in PBS for 1 h. Primary antibodies were added in blocking buffer at 1:500 concentration at 4 °C overnight. Secondary antibodies were added for 1 h at room temperature in blocking buffer. DAPI (Sigma-Aldrich) was used to stain cell nuclei.

Confocal imaging was done on Quorum Technologies WaveFx spinning-disk microscope set up on Olympus IX81 inverted stand (Olympus). Images were acquired using 60 ×, 1.42 numerical aperture oil objective, electron multiplying charge-coupled device (EM-CCD) camera (Hamamatsu) and Volocity software (PerkinElmer). Antibody information and co-localization analysis can be found in Supplemental Materials and Methods.

### Glucose uptake and lactate production

Cells were plated in complete growth medium for 48 h prior to glucose uptake measurement using the Amplex Red Glucose/Glucose Oxidase Assay Kit (Invitrogen). Lactate production was measured similarly, but after 96 h of growth in complete medium. Amplex Red Reagent (Invitrogen), lactate (MilliporeSigma, Burlington, MA, USA), and lactate oxidase (MilliporeSigma) were used to determine lactate concentration in the media. Fluorescence was measured at 590 nm. Background fluorescence was corrected for by subtracting the value of the control well. Fluorescence readings were then normalized to cell number.

### High-resolution respirometry and enzyme activity assays

Cells were grown in normal growth conditions with complete medium for 3 days. To measure hexokinase activity, Hexokinase Colorimetric Assay Kit (MilliporeSigma) was used as per manufacturer’s instructions. Absorbance was normalized to cell number. To measure citrate synthase and complex I activity, cells were lysed at −80 °C. Citrate synthase activity was measured at 412 nm recording the linear reduction of 0.1 mM 5,5′-dithiobis-2-nitrobenzoic acid in the presence of 0.31 mM acetyl-CoA, 0.5 mM oxalacetic acid, 0.1 M Tris/HCl, 50 μm EDTA, 5 mM triethanolamine hydrochloride (pH 8.1) [52]. Complex I activity was measured at 540 nm recording the linear oxidation of 0.10 mM NADH in the presence of 60 μM ubiquinone, 30 mM sodium azide, 50 mM phosphate buffer (pH 7.5), 3 mg/mL bovine serum albumin, and 0.5 μM rotenone in the blank (slight modification from [53–56]). Detailed methods for high-resolution respirometry can be found in Supplemental Materials and Methods.

### Statistical analysis

All statistical analysis was performed using GraphPad Prism Software. For comparisons between two groups, a Student’s *t*-test was used. For comparisons between greater than two groups, a one-way ANOVA followed by a Dunnett’s multiple comparisons test to compare all groups to a reference control, or a Tukey’s multiple comparisons test to compare all groups to each other was used. All data are presented as ± standard error of the mean (SEM). Experiments were performed at least three times. Statistical significance refers to *P < 0.05, **P < 0.01, ***P < 0.001, ****P < 0.0001.

## Supplementary information

Supplemental Materials and Methods

Supplemental Figure 1

Supplemental Figure 2

Supplemental Figure 3

Supplemental Figure 4

Supplemental Figure 5

Supplemental Figure 6
