# Supplementary material for: Non-canonical BAD activity regulates breast cancer cell and tumor growth via 14-3-3 binding and mitochondrial metabolism
Source: Oncogene. 2019 Jan 11;38(18):3325–39. doi: 10.1038/s41388-018-0673-6 (PMC6756016; doi:10.1038/s41388-018-0673-6)
Supplement: Supplementary file 1 — Supplemental Materials and Methods [file 41388_2018_673_MOESM1_ESM.docx]

**Supplemental Materials and Methods**

**2D Gel Electrophoresis**

For 2D-electrophoresis, cells were lysed using 2-dimension lysis buffer (7 M Urea, 2 M Thiourea, 4% CHAPS, and 30 mM Tris-HCl, pH 8.7). Protein quantification was performed using the 2-D Quant Kit (GE Healthcare, Little Chalfont, UK). 150µg of protein was supplemented with rehydration buffer (7M urea, 2M thiourea, 2% CHAPS, and 0.002% bromophenol blue) and incubated with an Immobiline DryStrip Gel (GE Healthcare) at room temperature for 20 hours. The first dimensional electrophoresis was performed on the IPGphor isoelectric focusing system (GE Healthcare). The strips were then equilibrated by first incubating in Buffer 1 (6M urea, 30% glycerol, 2% SDS, 75mM Tris-HCl, pH 8.8, and 1% DTT) for 15 minutes, and then in Buffer 2 (6M urea, 30% glycerol, 2% SDS, 50mM Tris-HCl, pH 8.8, and 2.5% iodoacetamide) for 15 minutes. The second dimensional electrophoresis was performed on the Mini-PROTEAN II system (Bio-Rad, Hercules, CA, USA). After equilibration, strips were placed on top of 12% SDS-PAGE gels and sealed using melted agar containing SDS-PAGE loading dye. A constant power of 1W/gel was applied before transfer to nitrocellulose membranes and subsequent immunoblot.

**High-Resolution Respirometry Analysis of Oxygen Flux**

Complex I substrates malate and pyruvate (M and P; 2 and 5 mM, respectively), malate and glutamate (M and G; 2 and 10 mM respectively), or Palmitoyl-CoA, malate and carnitine (Pal-Coa, M and C; 5, 5 and 25 mM respectively) were added and the LEAK state was initiated by permeabilization of the plasma membrane with addition of digitonin (Dig; 3.0 μg/mL).

Cell respiration was measured at 37 °C with the Oxygraph-2k (Oroboros Instruments, Innsbruck, Austria) in 2-mL chambers with a stirrer speed of 750 rpm. Data acquisition was performed using the software DatLab (Oroboros Instruments, Innsbruck, Austria). Automatic instrumental background corrections were applied for oxygen consumption by the polarographic oxygen sensor and oxygen diffusion into the chamber.

*Intact Cells*

The protocol for the respiration in intact cells is illustrated in Figure 6E. Respiration of intact cells (1x10^6^ cells/mL) was measured in RPMI 1640 culture medium (10% fetal calf serum) (Life Technologies) under cellular routine conditions (ROUTINE). After inhibition of ATP-synthase with 2 μg/mL oligomycin, respiration declined to the resting or leak-compensating state (LEAK state). Uncoupling with stepwise titration to an optimal concentration of the protonophore carbonyl cyanide p-(trifluoromethoxy) phenylhydrazone, FCCP, induced maximal noncoupled respiration as a measure of electron transfer system capacity (ET state). Residual oxygen consumption (ROX) was obtained after inhibition of CI and CIII with 0.5 μM rotenone and 2.5 μM antimycin A. Fluxes in all states were corrected for ROX and expressed per million cells.

*Plasma Membrane Permeabilization*

The optimum digitonin concentration for selective permeabilization of the plasma membrane was determined in vector and WT cells. ROUTINE respiration as supported by endogenous substrates was measured in mitochondrial respiration medium MIR05 (110 mM sucrose/60 mM K-lactobionate/0.5 mM EGTA/1 g/L BSA fatty acid free/3 mM MgCl_2_/20 mM taurine/10 mM KH_2_PO_4_/20 mM K-HEPES/pH 7.1). After addition of rotenone (0.5 μM), succinate (10 mM), and ADP (2.5 mM), digitonin was titrated in steps of 1 μg/mL.

*Permeabilized Cells*

After adding intact cells for measurement of ROUTINE respiration in MIR05 and permeabilization of cell membranes with optimal digitonin concentration (3.0 μg/mL), three different protocols were performed. The first protocol used (final concentration in the chamber) pyruvate (5 mM) and malate (5 mM) as NADH-linked substrates through Complex I, followed by ADP (2.5 mM), cytochrome c (c; 10 μM); succinate (CII-linked substrate; 10 mM), FCCP (titration to optimum concentration); rotenone (0.5 μM) and antimycin A (2.5 μM) as CI and CIII inhibitors; ascorbate (0.5 mM) and TMPD (2 mM) as Complex IV-linked substrates, and azide (100 mM) as a Complex IV inhibitor. In the second and third protocol, pyruvate and malate were replaced respectively by glutamate (10 mM) and malate (2 mM) or by palmitoyl-CoA (5 mM), carnitine (25 mM) and malate (5mM).

**BAD Knockdown in MDA-MB-231**

MDA-MB-231 cell lines stably expressing pcDNA3.2/V5-DEST vector were transfected with siRNA as previously described (1). Briefly, cells were plated for approximately 18 hours prior to addition of siRNA duplexes with HiPerFect Transfection Reagent (Qiagen): BAD siRNA target sequence: ACGAGTTTGTGGACTCCTTTA (Qiagen S100299348) or

AllStars Negative Control siRNA (Qiagen 1027280). Cells were incubated with siRNA for 12 hours prior to western blot analysis and for 96 hours prior to cell counts.

**BAD Knockdown and Cell Count Assay in MCF10A**

MCF10-A cells were from ATCC. Cells were grown in DMEM/F12 medium (Invitrogen) supplemented with 5% horse serum (Invitrogen), 20 ng/mL EGF (Cedarlane, Burlington, ON, CAN), 0.5 mg/mL hydrocortisone (Fisher Scientific, Ottawa, ON, CAN), 100 ng/mL cholera toxin (Cedarlane), and 10 μg/mL insulin (MilliporeSigma) at 37°C and 5% CO_2_. BAD knockdown in MCF10-A cells was generated using CompZr® Zinc Finger Nuclease (ZFN) Technology (MilliporeSigma). Two independent BAD knock-out clonal cell lines were created using nucleofection. “BAD Knockout 1” was generated using Zinc Finger Nuclease mRNA. TransIT®-mRNA Transfection Reagent (Mirus Bio, Madison, WI, USA) was used for transfection of the Zinc Finger Nuclease mRNA. “BAD Knockout 2” was generated using Zinc Finger Nuclease DNA. MCF10A control 1 and 2 are negative control cell lines that were subject to the same conditions to generate the knock-out cell lines and retained BAD expression. The following Zinc Finger Nuclease primers were used for the Zinc Finger Nuclease DNA:

Primer Forward: AGGGGCTTAGAGGAGCTGAG

Primer Reverse: AAGGAACAGGACGGCTTTG

The ZFN binding site (capital letters) and cutting site (lower-case letters) was as follows:

CTGGGCAGCACAgCGCTATggccgCGAGCTCCGGAGGATGAG

To test proliferation of mutants, 1x10^5^ cells were plated in a 6-well plate in complete medium and counted daily for 6 days using a hemocytometer.

**H&E Staining, Imaging and Analysis of Tumor Necrotic Area**

Tumor sections were dewaxed in xylene, then re-hydrated in decreasing amounts of ethanol then stained in Harris hematoxylin solution (Fisher Scientific) for 20 minutes. After rinsing in tap water, slides were differentiated in 1% acid alcohol (1% HCl in 70% ethanol) for 5 seconds, followed by incubation in Scott’s tap water (0.2% sodium bicarbonate, 2% magnesium sulphate) for blueing. Slides were washed in running tap water for 5 minutes and then rinsed in 95% alcohol briefly. Counterstain was performed in 1% eosin B (MilliporeSigma) solution for 1 minute. Slides were then dehydrated with increasing amounts of ethanol and cleared in two changes of xylene. Slides were mounted with Permount Mounting Medium (Fisher Scientific).

Tile imaging (10% overlap) of H&E stained tumors was done on Zeiss AxioObserver Z1 Microscope, 10x objective lens. Tiles were stitched together using a Fourier Shift Theorem based Fiji plugin (https://www.ncbi.nlm.nih.gov/pmc/articles/PMC2682522/ ). Image analyses was performed in MATLAB (MathWorks) to determine tumor region and necrotic areas. First, the H&E image colors were automatically segmented through L*a*b* color space and K-means clustering. Manual inspection of each image was done to ensure correct boundary detection.

**Immunofluorescence Co-localization Analysis**

For colocalization analysis, MATLAB (MathWorks) and ImageJ’s Coloc 2 plugin (http://imagej.net/Coloc_2) were used. The Java package MIJ/Miji (http://bigwww.epfl.ch/sage/soft/mij/) (2) was used enabling bi-directional communication and data exchange between MATLAB and ImageJ. In brief, cell boundary (region of interest-ROI) and background subtraction was computed as previously detailed using MATLAB (3). Manders' Colocalization Coefficients (MCC) were calculated using the Coloc 2 plugin with Costes' auto-threshold within the ROI (4). MCC represent fractions of proteins colocalizing with each other. To ensure that the measured colocalization was not obtained by chance, within the ROI, we scrambled blocks of pixels (4x4 pixels) of one channel (BAD protein channel) per iteration, repeated MCC measurements and used the mean MCC from 20 iterations. A schematic representation of this methodology is depicted (Supplemental Figure 6A).

**Mammosphere Formation and Analysis**

MDA-MB-231 cells expressing vector and wild-type BAD single cells were plated in replicates or triplicates in Poly-HEMA coated 24-well plates at 5 cells/mm3 suspension (500 μL suspension volume per well) in DMEM/F12 medium (1:1) supplemented with 20 ng/mL FGF-2, 20ng/mL EGF, 2% B27 without vitamin A, 1x ITS (insulin-transferrin-selenium) and 0.5% Methylcellulose. Every 3 days, 500 μL of fresh medium was added to each well without removing the old medium. Mammospheres were imaged in brightfield (Zeiss AxioObserver Z1 Microscope) on days 7, 14 and 21. Mammosphere formation efficiency was determined on day 21. It was calculated from number of spheres per well, divided by number of cells plated, multiplied by 100 (to convert it to percentage).

Image analyses was performed in MATLAB (MathWorks). To segment mammospheres in bright field images and determine their areas, a binary gradient image mask of the mammosphere(s) was calculated from a threshold value determined by edge and Sobel operator. Linear gaps in the gradient images were dilated using linear structuring elements, interior holes filled and resultant mammosphere boundary smoothened by eroding the image twice with a diamond structuring element. Manual inspection of each image was done to ensure correct boundary detection. A representative mammosphere boundary detection is depicted in Supplemental Figure 6B.

**Supplemental Tables**

**Supplemental Table S1. List of Primers**

| **Primer** | **Sequence** |
| --- | --- |
| hBAD forward | CACCATGTTCCAGATCCCAGAGTTTG |
| hBAD reverse | AAGCTTCACTGGGAGGGGGCGGAGCTT |
| hBAD S118A forward | CGAGCTCCGGAGGATGGCTGACGAGTTTGTGGAC |
| hBAD S118A reverse | GTCCACAAACTCGTCAGCCATCCTCCGGAGCTCG |
| hBAD S118D forward | CGAGCTCCGGAGGATGGATGACGAGTTTGTGGAC |
| hBAD S118D reverse | GTCCCAAACTCGTCATCCATCCTCCGGAGCTCG |
| hBAD S99A forward | CCGCTCGCGCGCGGCGCCCCC |
| hBAD S99A reverse | GGGGGCGCCGCGCGCGAGCGG |
| hBAD S99D forward | GGGCCGCTCGCGCGATGCGCCCCCCAACC |
| hBAD S99D reverse | GGTTGGGGGGCGCATCGCGCGAGCGGCCC |
| hBAD L114A forward | GCTATGGCCGCGAGGCCCGGAGGATGAGTG |
| hBAD L114A reverse | CACTCATCCTCCGGGCCTCGCGGCCATAGC |

**Supplemental Table S2. List of Antibodies**

| **Primary Antibody** | **Species** | **Reference** | **Application** |
| --- | --- | --- | --- |
| BAD | Rabbit | B0684 (MilliporeSigma) | WB, IP, IF |
| Tubulin | Mouse | T5168 (MilliporeSigma) | WB |
| pBAD Ser136 | Rabbit | 5286 (Cell Signaling Technologies) | WB |
| pAKT | Rabbit | 4058 (Cell Signaling Technologies) | WB |
| AKT | Rabbit | 9272 (Cell Signaling Technologies) | WB |
| Bcl-XL | Rabbit | 2762 (Cell Signaling Technologies) | WB, IP |
| pan 14-3-3 | Rabbit | sc-629 (Santa Cruz) | WB |
| Hexokinase I | Rabbit | C35C4 (Cell Signaling Technologies) | WB |
| Hexokinase II | Rabbit | C64G5 (Cell Signaling Technologies) | WB |
| Tom 20 | Rabbit | sc-11415 (Santa Cruz) | WB |
| Complex I | Mouse | A-21360 (Molecular Probes) | WB |
| pBAD-S118 | Rabbit | NB100-92481 (Novus Biologicals) | IP |
| GST | Rabbit | G7781 (MilliporeSigma) | IP |
| Calnexin | Mouse | 610523 (BD Biosciences) | IF |
| BAD | Rabbit | MA5-14800 (ThermoFisher Scientific) | IHC |
| pBAD-Ser136 | Rabbit | sc-7999 (Santa Cruz) | IHC |
| Ki-67 | Rabbit | 9027 (Cell Signaling Technologies) | IHC |
| Cleaved caspase-3 | Rabbit | 9664 (Cell Signaling Technologies) | IHC |
| Cleaved PARP | Rabbit | 9541 (Cell Signaling Technologies) | IHC |
| CD-31 | Rabbit | ab28364 (Abcam) | IHC |
| **Secondary Antibody** | **Species** | **Reference** | **Application** |
| Rabbit IgG | Goat | 1706515 (Bio-Rad) | WB |
| Mouse IgG | Goat | 1721011 (Bio-Rad) | WB |
| Rabbit IgG H&L | Goat | A-11034 (ThermoFisher Scientific) | IF |
| Mouse IgG H&L | Donkey | A-31571 (ThermoFisher Scientific) | IF |

**References**

1. Czernick M, Rieger A, Goping IS. Bim is reversibly phosphorylated but plays a limited role in paclitaxel cytotoxicity of breast cancer cell lines. Biochem Biophys Res Commun. 2009;379(1):145-50.

2. Hiner MC, Rueden CT, Eliceiri KW. ImageJ-MATLAB: a bidirectional framework for scientific image analysis interoperability. Bioinformatics. 2017;33(4):629-30.

3. Githaka JM, Vega AR, Baird MA, Davidson MW, Jaqaman K, Touret N. Ligand-induced growth and compaction of CD36 nanoclusters enriched in Fyn induces Fyn signaling. J Cell Sci. 2016;129(22):4175-89.

4. Costes SV, Daelemans D, Cho EH, Dobbin Z, Pavlakis G, Lockett S. Automatic and quantitative measurement of protein-protein colocalization in live cells. Biophys J. 2004;86(6):3993-4003.
