# Supplementary material for: Non-canonical BAD activity regulates breast cancer cell and tumor growth via 14-3-3 binding and mitochondrial metabolism
Source: Oncogene. 2019 Jan 11;38(18):3325–39. doi: 10.1038/s41388-018-0673-6 (PMC6756016; doi:10.1038/s41388-018-0673-6)
Supplement: Supplementary file 2 — Supplemental Figure 1 [file 41388_2018_673_MOESM2_ESM.pdf]

SUPPLEMENTAL FIGURE 1

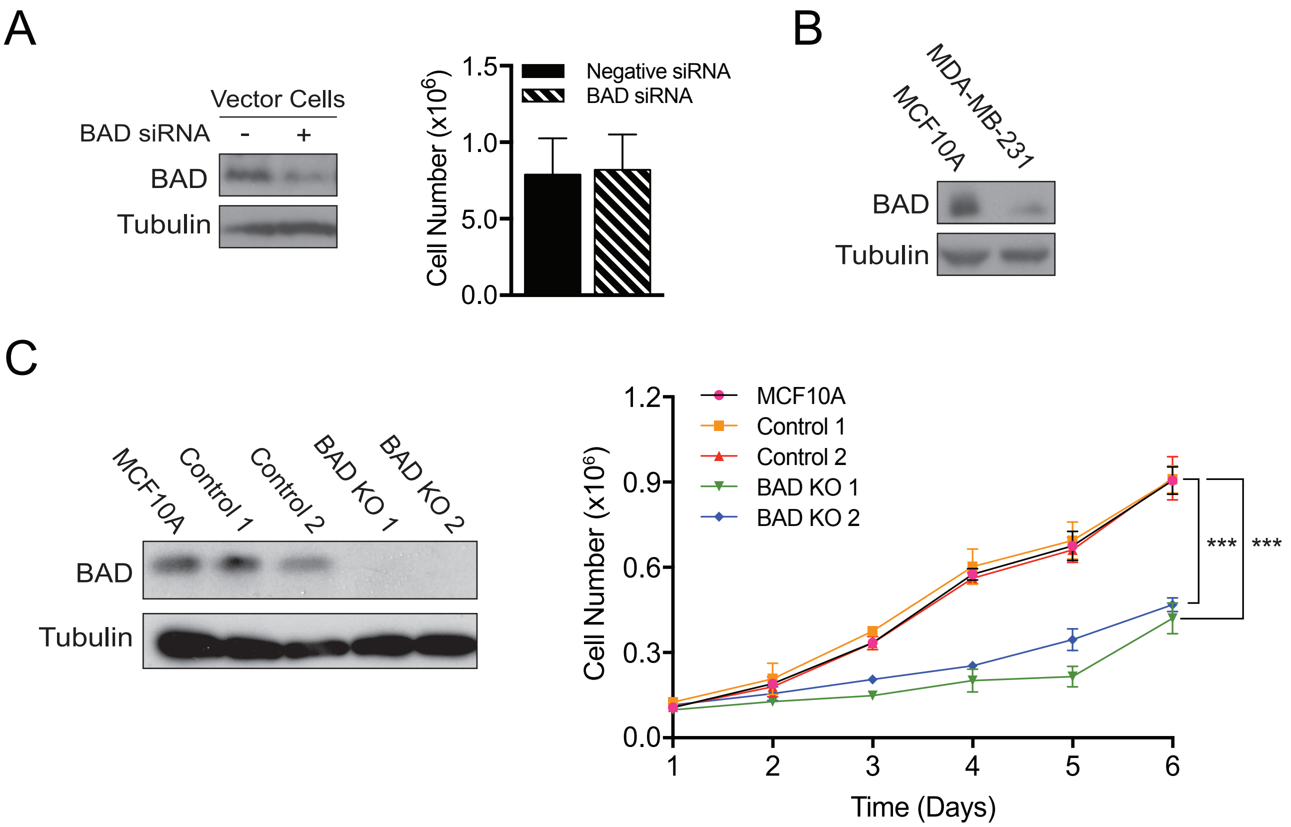

Supplemental Figure 1. BAD expression maintains cellular survival in normal breast epithelial cells

(A) Left: Western bot verifying BAD knockdown in MDA-MB-231 vector cells. Right: Cell counts at 96 hours post-transfection with siRNA duplexes for BAD or negative control (no significance; error bars ± SEM). (B) Western blot comparing endogenous BAD expression of the indicated parental cell lines. (C) Left: Two independent BAD knock-out clonal cell lines were generated using CompZr® Zinc Finger Nuclease Technology. Tubulin was used as a loading control. Right: $1x10^5$ cells were plated in duplicate in a 6-well plate in complete medium and counted daily for 6 days using a hemocytometer (error bars ± SEM of 3 independent experiments).
