# Supplementary material for: Non-canonical BAD activity regulates breast cancer cell and tumor growth via 14-3-3 binding and mitochondrial metabolism
Source: Oncogene. 2019 Jan 11;38(18):3325–39. doi: 10.1038/s41388-018-0673-6 (PMC6756016; doi:10.1038/s41388-018-0673-6)
Supplement: Supplementary file 3 — Supplemental Figure 2 [file 41388_2018_673_MOESM3_ESM.pdf]

SUPPLEMENTAL FIGURE 2

A

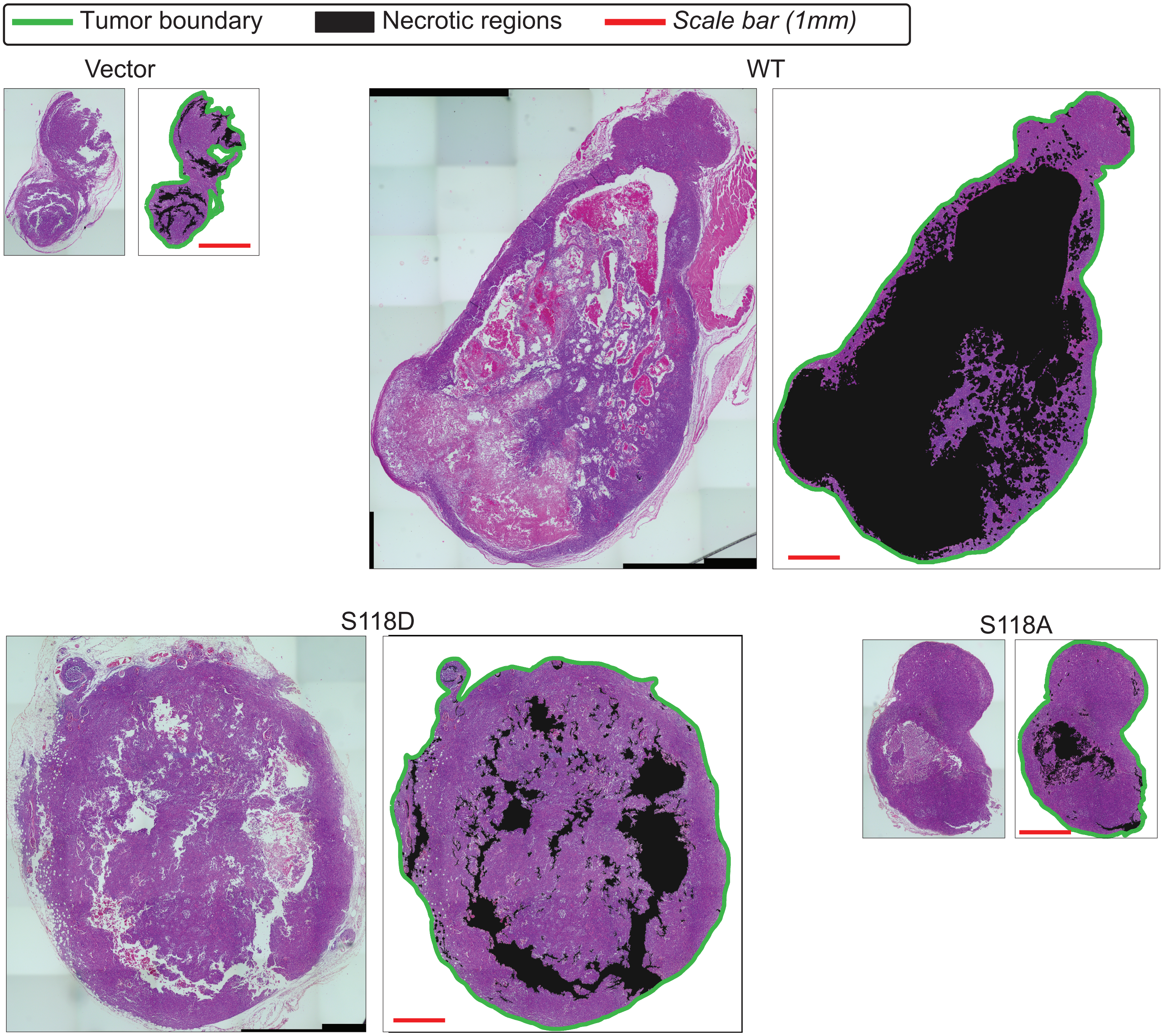

B

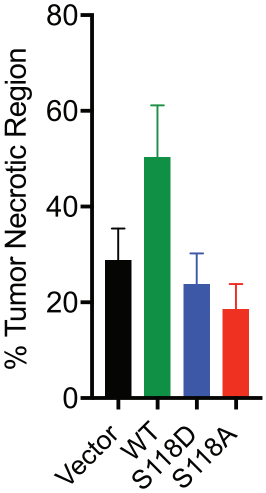

Supplemental Figure 2. Wild-type BAD tumors depict large areas of necrosis (A) Representative images of H&E stained tumor sections of subcutaneous xenograft tumors, derived from MDA-MB-231 cells expressing pcDNA3.2-V5-DEST vector control, WT-BAD, BAD-S118D, and BAD-S118A. The corresponding boundary detection (green line) and necrotic regions (black regions) are indicated. All images are sized to the same scale (red line scale bar=1 mm). (B) Percentage of necrosis per total tumor area was calculated. The number of tumors analyzed per group were as follows: vector n=4, WT-BAD n=4, BAD-S118D n=6, and BAD-S118 n=2 (no significance; error bars ± SEM).
