# Supplementary material for: Non-canonical BAD activity regulates breast cancer cell and tumor growth via 14-3-3 binding and mitochondrial metabolism
Source: Oncogene. 2019 Jan 11;38(18):3325–39. doi: 10.1038/s41388-018-0673-6 (PMC6756016; doi:10.1038/s41388-018-0673-6)
Supplement: Supplementary file 4 — Supplemental Figure 3 [file 41388_2018_673_MOESM4_ESM.pdf]

SUPPLEMENTAL FIGURE 3

A

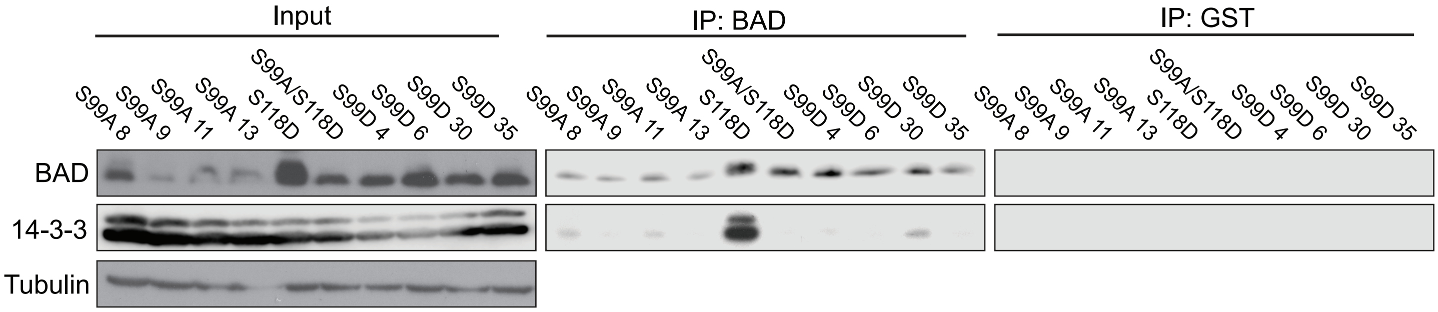

B

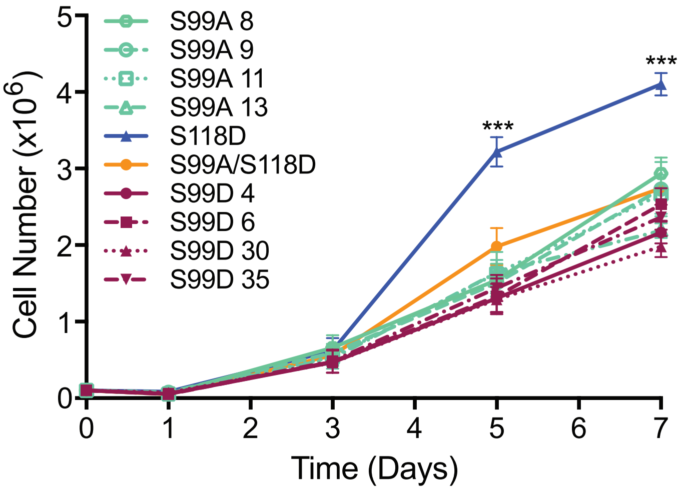

Supplemental Figure 3. 14-3-3 binding is required for BAD-S118D mediated cell growth
(A) MDA-MB-231 cells expressing the indicated mutations were subjected to immunoprecipitation by BAD or GST (control) antibodies and immunoblotted against BAD, 14-3-3, and Tubulin. (B) Cell count assay over 7 days (all groups compared to S118D; error bars ± SEM of 3 independent experiments).
