# Supplementary material for: Non-canonical BAD activity regulates breast cancer cell and tumor growth via 14-3-3 binding and mitochondrial metabolism
Source: Oncogene. 2019 Jan 11;38(18):3325–39. doi: 10.1038/s41388-018-0673-6 (PMC6756016; doi:10.1038/s41388-018-0673-6)
Supplement: Supplementary file 5 — Supplemental Figure 4 [file 41388_2018_673_MOESM5_ESM.pdf]

SUPPLEMENTAL FIGURE 4

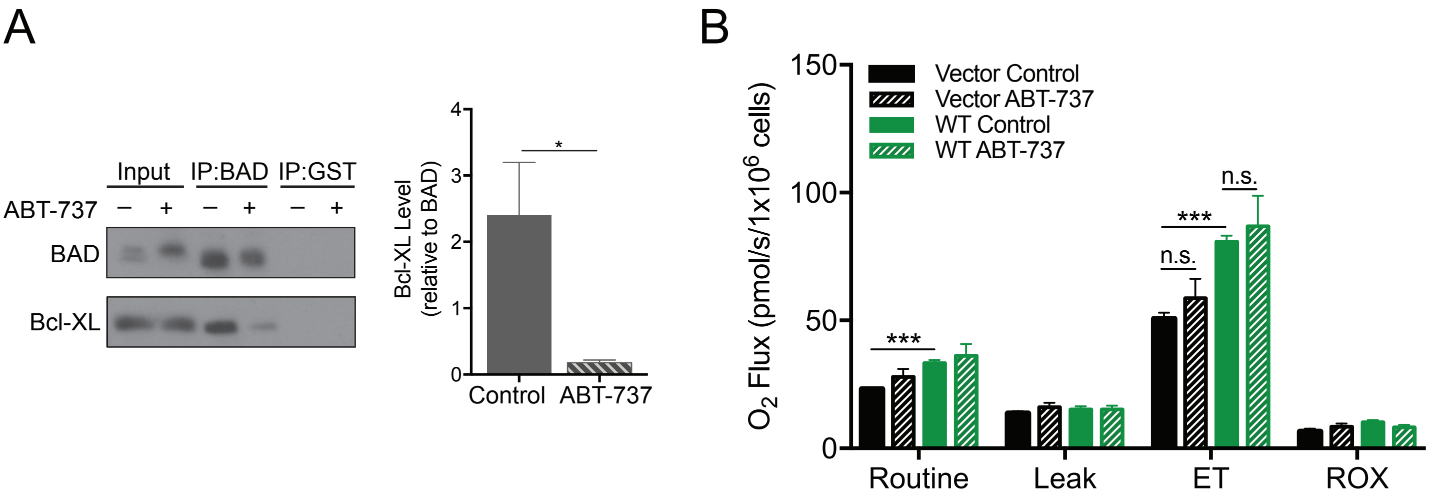

Supplementary Figure 4. The BAD:Bcl-XL interaction is not required for BAD mediated mitochondrial metabolism

(A) Left: MDA-MB-231 cells expressing wild-type BAD were incubated with 20 μm ABT-737 for 24 hours prior to immunoprecipitation with BAD antibody to confirm Bcl-XL inhibition. Right: Quantification of Bcl-XL binding to BAD (error bars ± SEM of 3 independent experiments). (B) MDA-MB-231 cells expressing pcDNA3.2-V5-DEST vector control or wild-type BAD were grown for 48 hours in normal growth medium prior to 24 hour addition of 20 μm ABT-737. Cells were then subjected to high resolution respirometry (error bars ± SEM of 4 independent experiments).
