# Supplementary material for: Non-canonical BAD activity regulates breast cancer cell and tumor growth via 14-3-3 binding and mitochondrial metabolism
Source: Oncogene. 2019 Jan 11;38(18):3325–39. doi: 10.1038/s41388-018-0673-6 (PMC6756016; doi:10.1038/s41388-018-0673-6)
Supplement: Supplementary file 6 — Supplemental Figure 5 [file 41388_2018_673_MOESM6_ESM.pdf]

SUPPLEMENTAL FIGURE 5

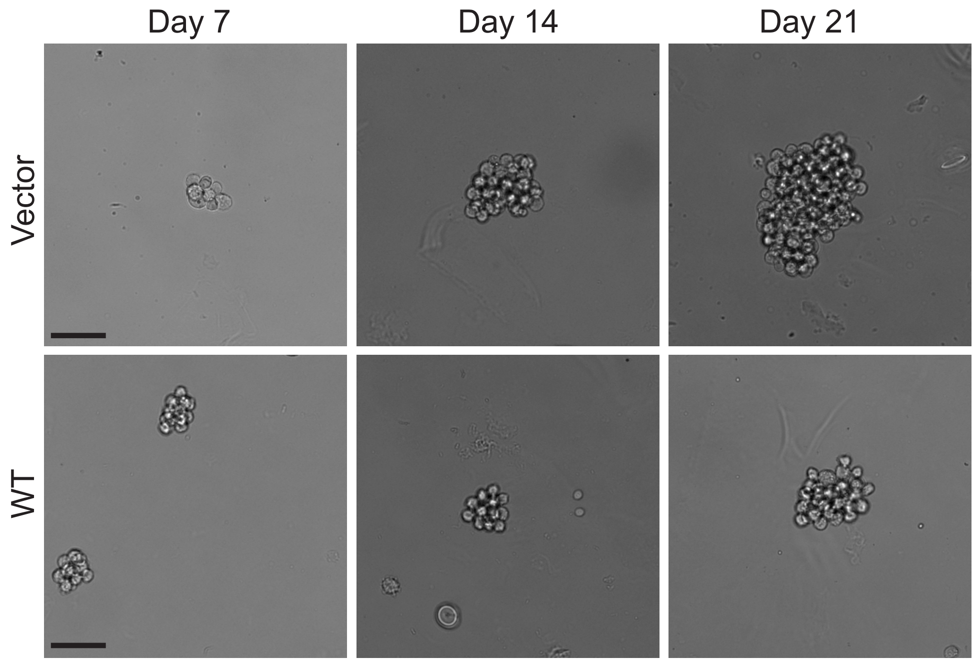

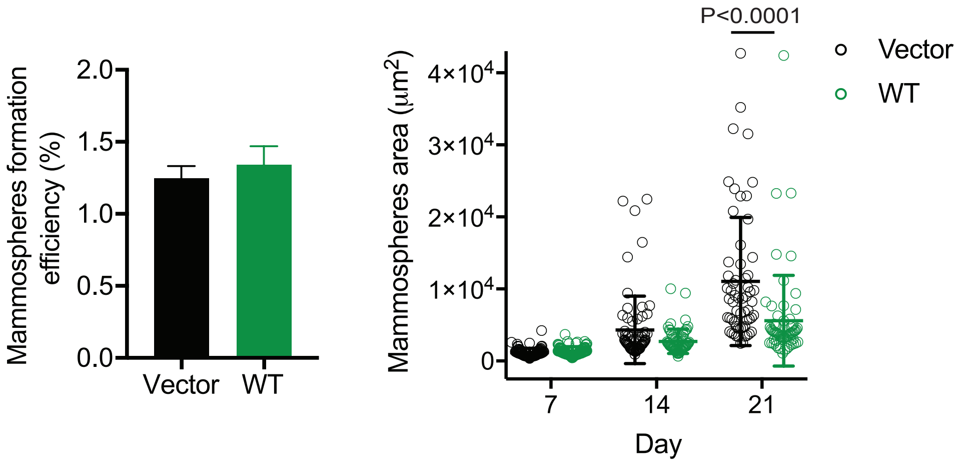

Supplemental Figure 5. BAD expression decreases mammosphere size
MDA-MB-231 vector and wild-type BAD single cells were grown in medium allowing for growth of mammospheres. Images were taken at the indicated day. Scale bar=50 μm. Mammosphere formation efficiency was measured on day 21 (error bars indicate SEM). Mammosphere area was measured on day 7, 14 and 21 (Two-way ANOVA followed by Sidak’s multiple comparisons test).
