# Supplementary material for: Non-canonical BAD activity regulates breast cancer cell and tumor growth via 14-3-3 binding and mitochondrial metabolism
Source: Oncogene. 2019 Jan 11;38(18):3325–39. doi: 10.1038/s41388-018-0673-6 (PMC6756016; doi:10.1038/s41388-018-0673-6)
Supplement: Supplementary file 7 — Supplemental Figure 6 [file 41388_2018_673_MOESM7_ESM.pdf]

SUPPLEMENTAL FIGURE 6

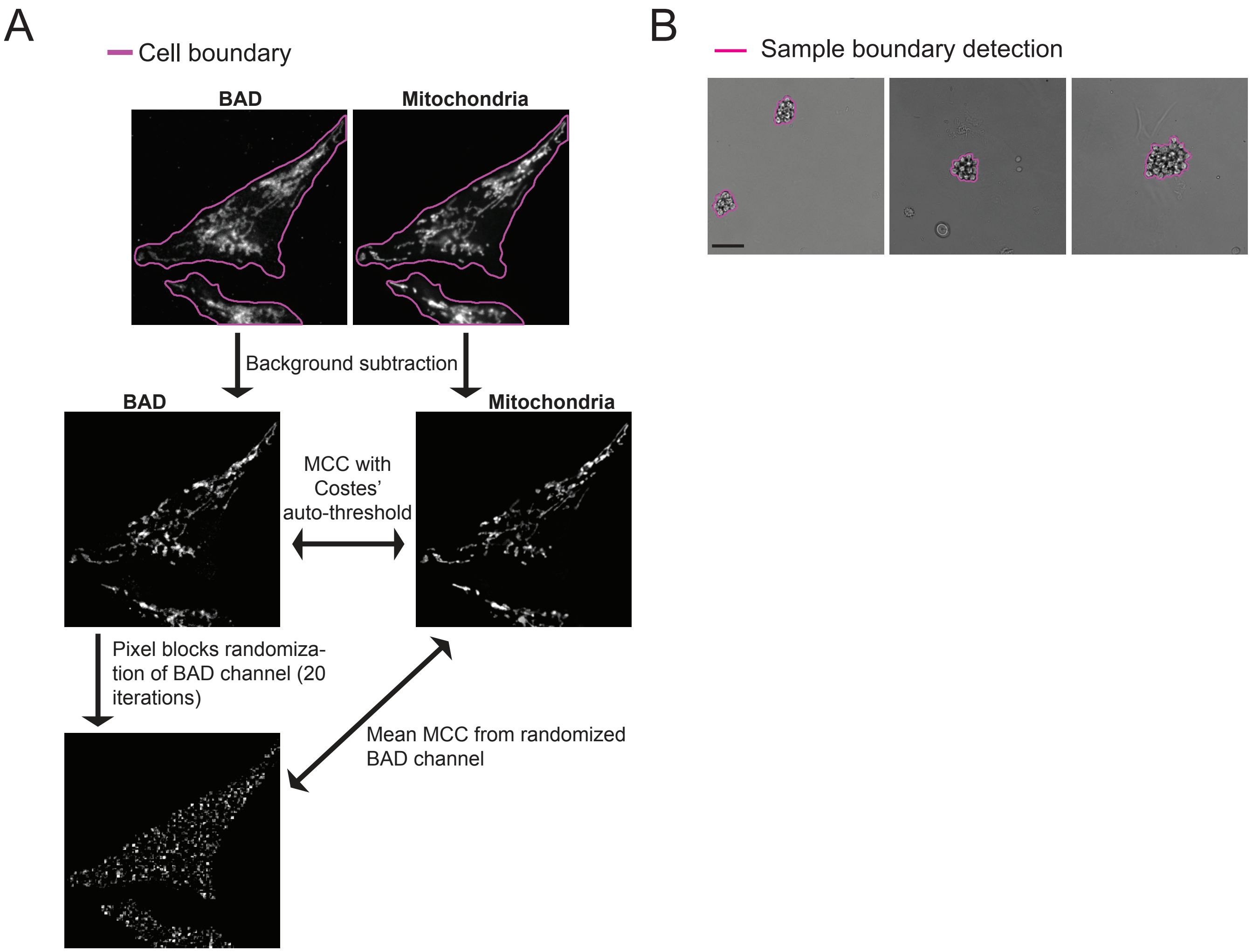

Supplemental Figure 6. Visual representation of immunofluorescence co-localization analysis and mammosphere area measurement

(A) Manders' Colocalization Coefficients (MCC) analysis flow (see Supplemental Materials and Methods). Analysis was done within the cell boundary (magenta line) determined from cell mask segmentation. Image background subtraction was followed by MCC computation using Costes' auto threshold. To ensure that the measured colocalization was not obtained by chance, pixel blocks of BAD protein channel were randomized within cell boundary and mean MCC from 20 iterations obtained. (B) Representative images of sample boundary detection of mammospheres. Scalebar=50 μm.
